# Ethical allocation of scarce vaccine doses: The Priority-Equality protocol

**DOI:** 10.3389/fpubh.2022.986776

**Published:** 2022-12-13

**Authors:** Carlos Alós-Ferrer, Jaume García-Segarra, Miguel Ginés-Vilar

**Affiliations:** ^1^Zurich Center for Neuroeconomics, University of Zurich, Zurich, Switzerland; ^2^Department of Economics, Universitat Jaume I, Castellón de la Plana, Spain

**Keywords:** rationing, vaccines, COVID-19, pandemics, medical ethics

## Abstract

**Background:**

Whenever vaccines for a new pandemic or widespread epidemic are developed, demand greatly exceeds the available supply of vaccine doses in the crucial, initial phases of vaccination. Rationing protocols must then fulfill a number of ethical principles balancing equal treatment of individuals and prioritization of at-risk and instrumental subpopulations. For COVID-19, actual rationing methods used a territory-based first allocation stage based on proportionality to population size, followed by locally-implemented prioritization rules. The results of this procedure have been argued to be ethically problematic.

**Methods:**

We use a formal-analytical approach arising from the mathematical social sciences which allows to investigate whether any allocation methods (known or unknown) fulfill a combination of (ethical) desiderata and, if so, how they are formulated algorithmically.

**Results:**

Strikingly, we find that there exists one and only one method that allows to treat people equally while giving priority to those who are worse off. We identify this method down to the algorithmic level and show that it is easily implementable and it exhibits additional, desirable properties. In contrast, we show that the procedures used during the COVID-19 pandemic violate both principles.

**Conclusions:**

Our research delivers an actual algorithm that is readily applicable and improves upon previous ones. Since our axiomatic approach shows that any other algorithm would either fail to treat people equally or fail to prioritize those who are worse off, we conclude that ethical principles dictate the adoption of this algorithm as a standard for the COVID-19 or any other comparable vaccination campaigns.

## 1. Introduction

When vaccines for the COVID-19 pandemic first became available, countries around the world rushed to secure as many doses as possible, with little or no attempt at coordination (1–5). To mitigate the ensuing chaos, and to provide global, equitable access to vaccines, the World Health Organization (WHO), the Coalition for Epidemic Preparedness Innovations (CEPI), and the Vaccine Alliance (Gavi) proposed the multilateral initiative Covax Facility (COVAX). The European Union also acted on behalf of all its member states (6), and the U.S. allocated vaccines among jurisdictions (7). In those and many other cases, vaccines were centrally acquired and had to be distributed among the territories.

The allocation of a centrally-acquired stock of (scarce) medical resources to individual territories is, however, a complex problem which is encountered in different environments. In the case of COVID-19, the various organizations referred above implemented different procedures on the basis of their political support rather than objective, scientific criteria (8). As a result, an ethical debate erupted, pointing out a number of serious flaws in the implemented procedures (9, 10). For example, some territories received enough vaccines to start inoculating lower-priority groups while other territories were not able to fully immunize higher-priority ones (11, 12). Further, individuals noticed that they would in practice receive higher priority if they simply moved from one territory to the next (13, 14).

According to a prominent strain of the medical ethics literature (15–18), the key ethical desiderata (or “fundamental values”) to take into account when facing the rationing of scarce medical resources are four: “(i) maximizing the benefits produced by scarce resources, (ii) treating people equally, (iii) promoting and rewarding instrumental value, and (iv) giving priority to the worst off” [(17), p. 2051]. If we restrict our attention to the allocation of scarce vaccines, the first and the third fundamental values are relevant to establish the different priority classes. In other words, priority classes should be defined to reflect medical and scientific evidence as, for example, the different effects of treatments on individuals in different health states (12), and practical considerations as those dictating preferential treatment of medical personnel. In contrast, the challenges arising from the distributional aspects of vaccine allocation involve the second and the fourth fundamental values above.

In this contribution, we shed light on these distributional aspects of the allocation of scarce vaccines. In other words, we assume that the priority classes have already been decided (e.g., by experts in medical ethics and organizations like the WHO). The remaining key ethical desiderata are two. First, priority should be given to worse-off groups (as the elderly or otherwise at-risk) and to those with instrumental value (as, e.g., medical personnel). Second, equal people, that is, those with the same priority, should be treated equally, meaning that they should have the same likelihood of being vaccinated.

There exist many frameworks addressing distributional fairness in healthcare (4, 16–22), but they typically remain unspecified, that is, they discuss desiderata but stop short of identifying actual distribution mechanisms. For the case of vaccine allocation, at this point, society needs an allocation procedure or *vaccine rationing protocol* fulfilling the two key requirements discussed above. That is, higher-priority classes should receive vaccinations before lower-priority ones, and people within the same priority class should be treated equally.

**Figure 1:**
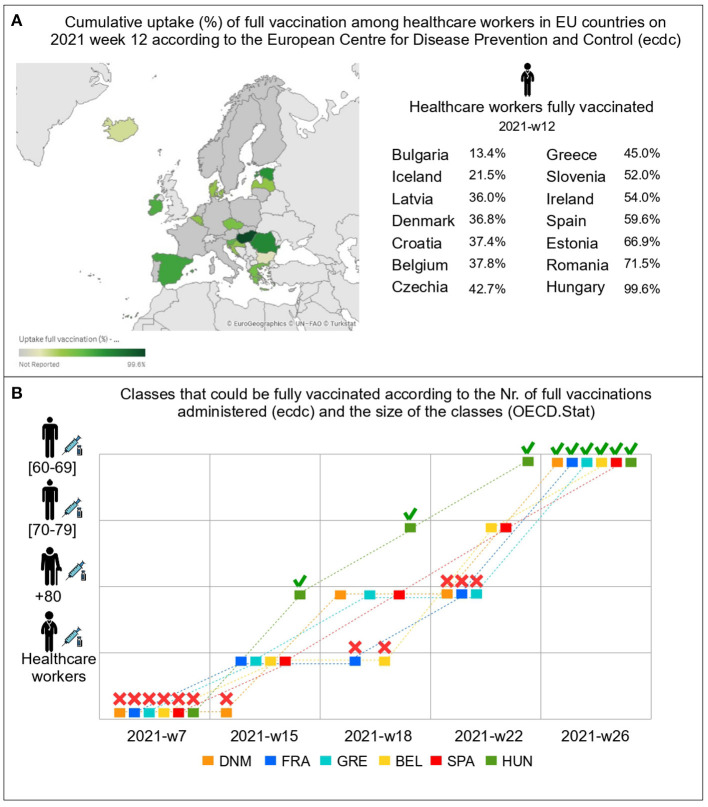
Data and map from the European Center for Disease Prevention and Control (ecdc) and OECD.Stat. **(A)** shows a high dispersion in the vaccination rates of healthcare workers within the EU in week 12/2021, showing that *Prioritizing According To Needs* was violated. **(B)** uses OECD data (Table 1) and shows which priority classes could have been fully immunized at different points in time (2021) according to ecdc data on full vaccinations administered (primary course) in each territory. This exercise shows that *Treating Equal People Equally* was violated. For example, on week 22*nd*, beyond the case of Hungary (which decided to buy vaccines from third countries on top of the agreement with the EU), we observe that Belgium and Spain could have started immunizing citizens from 60 to 69 years while France, Greece, or Denmark did not have enough vaccines to have immunized all citizens above 70 years. Icons made by Freepik from Flaticon.

The rationing protocols used to distribute vaccines in the case of COVID-19 failed on all accounts. Indeed, the dispersion in vaccination rates for given priority classes in EU countries was remarkable. In the 12th week of 2021, 99.6% of all healthcare workers were vaccinated (primary course) in Hungary,^1^ 71.5% in Romania, and 66.9% in Estonia, while the rates of fully-vaccinated health care workers were much lower in Denmark (36.8%), Czechia (42.7%), Ireland (54%), or Iceland (21.5%) (Figure 1A). Note that the vaccination rates for the healthcare workers in these countries were near 100% in January 2022, demonstrating that healthcare workers of those countries had a clear preference for being vaccinated, and hence that equal people were far from being treated equally.^2^

It is also clear that prioritization was violated. Figure 1B reports a simple exercise performed using publicly-available vaccination numbers from the European Center for Disease Prevention and Control and the size of population groups (healthcare workers and age groups) from the OECD (Table 1). Respecting priority means that, say, all healthcare workers in the entire EU should have been vaccinated (or it should have been possible to vaccinate them if willing) before immunization of other classes started, that no 70-year old should be vaccinated before all those aged above 80 were vaccinated in the entire EU, etc. In reality, in week 22, Spain and Belgium had received enough vaccines to vaccinate all healthcare workers and those aged 70 or older, while Denmark, France, and Greece only had received enough to immunize those older than 80, and Hungary already had enough to immunize all above 60. Even ignoring the case of Hungary (which had bought additional vaccines), this illustrates that the allocation protocol did not respect priority classes, as within the same vaccination alliance (the EU) people from lower-priority classes were being immunized while higher-priority classes had still not been fully vaccinated.

**Table 1:** Table reporting actual data about full vaccinations administered (primary course) at weeks 15, 18, 22, 26 of 2021 and the population by priority class according to the OECD.Stat.

	**Nr. of full vaccinations administered**				**OECD statistical data**			

**EU members**	**2021-w15**	**2021-w18**	**2021-w22**	**2021-w26**	**Healthcare W**.	**Age +80**	**Age [70–79]**	**Age [60–69]**
Hungary	1,362,623	2,460,201	3,929,646	4,958,419	318,508	441,525	867,394	1,282,913
France	4,534,880	7,875,751	12,630,071	22,593,343	3,899,000	4,137,718	5,823,662	8,002,188
Spain	3,447,162	6,278,419	10,956,826	19,121,368	1,527,500	2,856,102	3,986,744	5,427,445
Greece	769,325	1,215,279	2,306,762	3,966,764	266,627	774,369	1,012,738	1,292,835
Belgium	762,582	1,138,287	2,593,255	4,230,452	646,600	652,184	936,587	1,357,188
Denmark	488,572	848,769	1,351,828	2,094,482	525,000	277,977	570,642	664,621

We make use of the axiomatic approach proper of the mathematical social sciences. Formally, one translates ethical desiderata into analytical properties to be satisfied by protocols (themselves defined as mathematical functions), and then reverse-engineers the protocols fulfilling those properties. Very often, when using this methodology, one ends up with an impossibility theorem which reveals that the desiderata are mutually incompatible or too exacting. In this contribution, we demonstrate that the problem to find a vaccine allocation protocol fulfilling prioritization and equal treatment can be solved analytically. The failures illustrated above were not borne out of lack of competence at any level, or of inefficiency in administering a protocol. Rather, they are inherent failures of the protocols that were used. We show analytically that those protocols cannot fulfill the requirements of prioritization and equal treatment. We go even further and show that there exists one and only one protocol that satisfies both requirements. This means that *any other* protocol, already-existing or yet-to-be-designed, will either fail to respect priority classes or treat people within the same priority class unequally. This includes all protocols that have been used to distribute vaccinations for the COVID-19 pandemic. We pin down the new protocol to the algorithmic, ready-to-use level, and offer it for use by any vaccine alliance.

Intuitively, the protocol identified by our approach proceeds as follows. First, one must consider the size of the different priority classes *jointly across territories*, and distribute (on paper) vaccines to classes according to their priorities. Second, for each priority class, one distributes the vaccines to the territories proportionally to the size of the class in each territory. This protocol can be applied to any multi-territory organization, ranging from current multi-jurisdiction countries and alliances to the ideal of a global vaccine distribution (19, 20, 28–30). In contrast, currently-used protocols have been essentially based on an approach where, first, vaccines are allocated to territories proportionally to population (7, 31, 32), and then allocated to classes following priority within each territory (33, 34). As we show below, these methods violate both desiderata described above, and hence are inherently flawed, both technically and ethically.

## 2. Materials and methods

The formal-analytical proof of the existence and uniqueness result given below is in the Supplementary material.

## 3. Results

### 3.1. Vaccine rationing problems and ethical desiderata

There are *T* ≥ 2 territories, indexed by *t* = 1, …, *T*, and *P* priority classes, *p* = 1, …, *P*. A total of *V* > 0 vaccinations are available and must be allocated across territories and priority classes. For simplicity, a “vaccination” refers to a unit containing the number of doses required to immunize a single individual (e.g., two doses for most of the initial COVID-19 vaccinations, one for booster campaigns, etc.).

Territorial authorities report or request the number of vaccinations required for each class in each territory. Denote by $c_{t}^{p}$ the corresponding *claim* for class *p* in territory *t*. Let $c^{p}=c_{1}^{p}+\cdots +c_{T}^{p}$ and $c_{t}=c_{t}^{1}+\ldots +c_{t}^{P}$ be the total claims for class *p* and territory *t*, respectively. The total claim is $C=c^{1}+\ldots +c^{P}=c_{1}+\ldots +c_{T}$. A *Vaccine Rationing Problem* appears when *C*>*V*, that is, the total claim exceeds the available number of vaccinations and hence the latter must be rationed and distributed across territories and priority classes.

Let $n_{t}^{p}$ be the number of individuals belonging to priority class *p* in territory *t*, which is observable through administrative records. Denote by $n_{t}=n_{t}^{1}+\ldots +n_{t}^{P}$ the total population of territory *t*, and by *N* = *n*_1_ + … + *n*_*T*_ the total population across all territories. Obviously, claims are constrained so that $c_{t}^{p}\leq n_{t}^{p}$ for each *p* and *t*. To simplify notation, write *c* and *n* to denote the matrices containing all $c_{t}^{p}$ and all $n_{t}^{p}$, respectively.

The solution for Vaccine Rationing Problems is a *Vaccine Rationing Protocol* which takes as inputs the claims $c_{t}^{p}$ and the population sizes $n_{t}^{p}$, and delivers as outputs the vaccinations allocated to each *p* and *t*, denoted by *R*(*c, V*). This is in itself a matrix, that is, given claims $c_{t}^{p}$ and total available vaccinations *V*, the protocol *R* allocates $R_{t}^{p}\left(c,V\right)$ vaccinations to priority class *p* in territory *t*. The only requirement is that the $R_{t}^{p}\left(c,V\right)$ sum up to *V*, i.e., all vaccinations are allocated. Formally, every protocol *R* is a mathematical mapping between well-defined spaces (see Supplementary material A.1). The key of our contribution is that ethical desiderata can be translated into formal properties which such protocols should fulfill, and the axiomatic approach from the mathematical social sciences (see Supplementary material A.3) allows to pin down an exact protocol as the only one which satisfies the desiderata.

Among the ethical desiderata discussed in the introduction, the technical aspects of the requirement to maximize the benefits of scarce vaccinations are already encompassed in the definition of Vaccine Rationing Protocol, and specifically into the condition that the $R_{t}^{p}\left(c,V\right)$ add up to *V*, that is, no vaccinations are wasted. The requirement to promote and reward instrumental value means that priority classes should take into account professional groups as e.g., clinical or security personnel—since we take the priority classes as given, for our purposes this requirement is subsumed in the requirement to prioritize worse-off groups, which means that classes with higher priority (lower *p*) should receive vaccinations before the immunization of classes with worse priority starts.

Say that a class in a territory is *rationed* if it does not fully receive its claim, i.e., $R_{t}^{p}\left(c,V\right)<c_{t}^{p}$, and that it is *excluded* if it receives zero, i.e., $R_{t}^{p}\left(c,V\right)=0$. The requirement that priority classes are honored is the following.

**Prioritizing According To Needs**. If a class in a territory is rationed, then every class with worse priority (in *any* territory) is excluded.

Formally, this can be written as follows: If $R_{t}^{p}\left(c,V\right)<c_{t}^{p}$ for some *p* and some *t*, then $R_{t^{\prime }}^{p^{\prime }}\left(c,V\right)=0$ for all *p*′ = *p* + 1, …, *P* and for all *t*′. In other words, if there are not enough vaccinations to immunize all the individuals in a priority class for a given territory, then the protocol must not have allocated vaccinations to any lower-priority class in *any* territory. The requirement that this holds for any territory is crucial; as we will see below, standard allocation procedures during COVID-19 violated this desideratum.

The second desideratum is that (equal) people should be treated equally. Obviously, it is impossible to treat everybody equally while respecting priority of different classes, and what is actually meant by this desideratum is that two different people belonging to the same class should not face different constraints, and specifically should not be treated differently across territories. To formulate this principle, let the *vaccination rate* of a priority class be the proportion of people in the class that can be inoculated, given the number of allocated vaccinations, i.e., $R_{t}^{p}\left(c,V\right)/c_{t}^{p}$.

**Treating Equal People Equally**. The vaccination rates of priority classes do not differ across territories.

Formally, this means that, for every priority class *p* and any two territories *t, t*′,


$$\frac{R_{t}^{p}\left(c,V\right)}{c_{t}^{p}}=\frac{R_{t^{\prime }}^{p}\left(c,V\right)}{c_{t^{\prime }}^{p}}$$

provided $c_{t}^{p}>0$ and $c_{t^{\prime }}^{p}>0$.

The ratios can also be interpreted as the probability of being vaccinated for priority class and territory. Thus, this desideratum implies that individuals cannot increase their vaccination chances by moving from one territory to another.

### 3.2. Why COVID-19 protocols violated ethical desiderata

Generally speaking (as, e.g., in the case of COVAX or the EU), COVID-19 vaccinations were distributed in a two-stage fashion where first vaccinations were allocated to territories (31, 32), and then those allotments were redistributed across the priority classes in each territory (33, 34).

These naïve protocols violate both ethical principles, *Prioritizing According To Needs* and *Treating Equal People Equally*. To see this, we first define the procedure sketched above formally. This is a simplified procedure sharing the defining characteristics of the methods actually used in the case of COVID-19, and it suffices to see why such methods are problematic. For this purpose, we describe the procedure used by the European Union to allocate vaccines among their Member States proportionally to their population, with the simplifying assumption that in the second stage all Member States treat the priority classes the same way.

**Figure 2:**
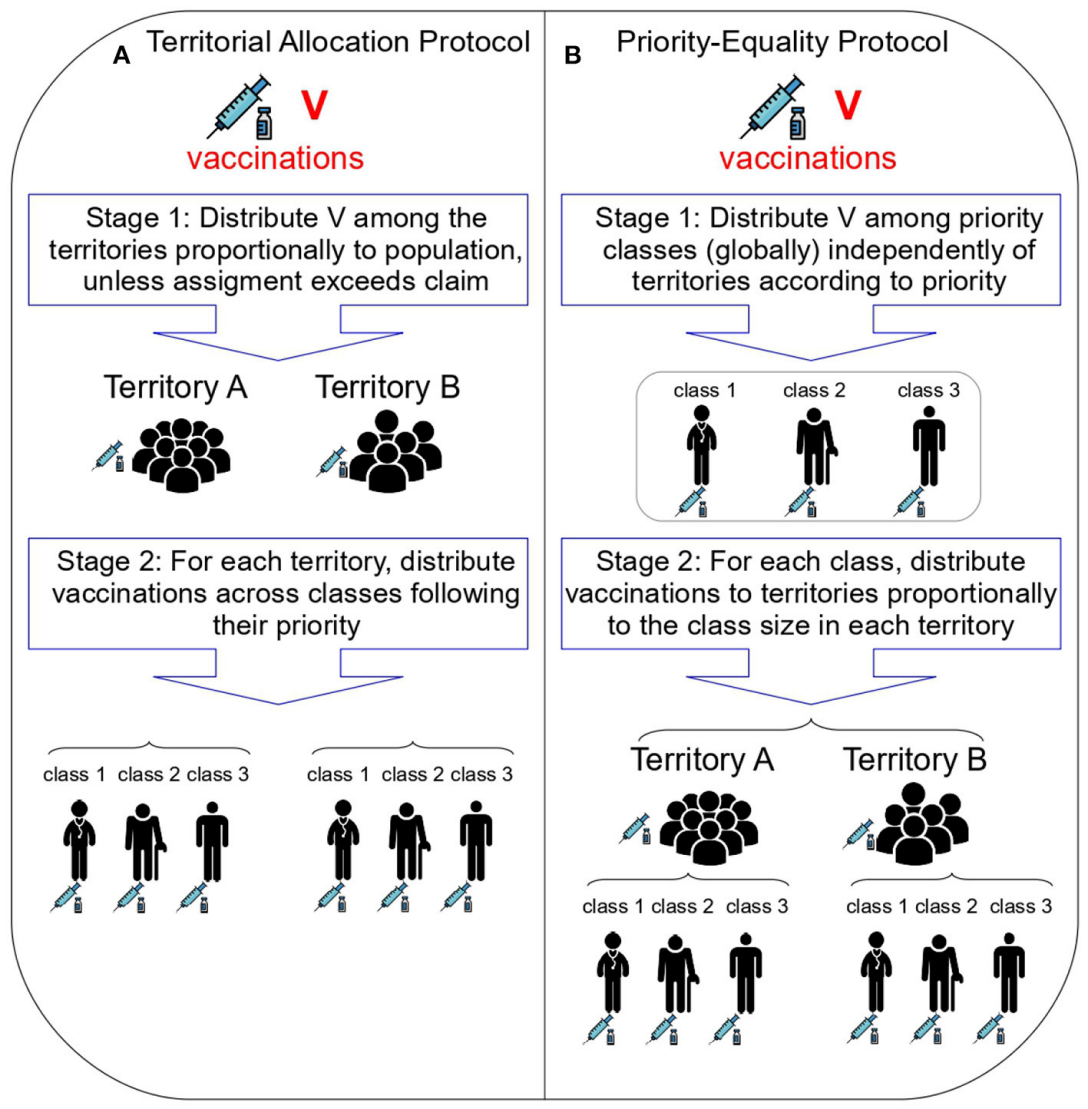
The Territorial Allocation and Priority-Equality protocols. **(A)** (left) shows the two stages of the Territorial Allocation protocol, which captures the essential features of those actually used for COVID-19. **(B)** (right) shows the two stages of the Priority-Equality protocol, which we identify in this work as the only one satisfying fundamental ethical principles. Icons made by Freepik from Flaticon.

**The Territorial Allocation protocol (TA)**. This protocol, which mimics those actually used for COVID-19 in a simplified way, proceeds as follows (Figure 2A). In the first stage, the available vaccinations *V* are distributed among the territories proportionally to the territories' population, unless the territorial claim can be fulfilled. Specifically, each territory *t* receives a total allotment of


$$R_{t}\left(c,V\right)=\min\left\{c_{t},\text{λ}\frac{n_{t}}{N}\right\}$$

where *N* = *n*_1_ + … + *n*_*T*_ is the total population across all territories and λ is a constant computed to guarantee that the vaccinations are exhausted, i.e.,


$$\underset{t=1\ldots ,T}{\sum }\min\left\{c_{t},\text{λ}\frac{n_{t}}{N}\right\}=V$$

In words, the procedure allocates *V* proportionally to the population of the territories, but reduces the allocation to the actual claim if that is exceeded, reallocating the excess vaccinations to other territories. Hence, λ is the proportion of the total population that can be vaccinated for those territories that are actually rationed.

In the second stage, the TA protocol distributes the allotment *R*_*t*_(*c, V*) of each territory *t* among the priority classes in *t* following the order of the priority classes. That is, for each territory, the highest-priority class is vaccinated, with the remaining vaccinations (if any) going to the second class, and so on until the doses are exhausted. Formally, let p^*^(*t*) be the unique class such that $c_{t}^{1}+\ldots +c_{t}^{p^{*}\left(t\right)-1}\leq R_{t}\left(c,V\right)$ but $c_{t}^{1}+\ldots +c_{t}^{p^{*}\left(t\right)}>R_{t}\left(c,V\right)$, i.e., the first class in order of decreasing priority such that available vaccinations in the territory do not allow for full coverage. Then,


$$R_{t}^{p}(c,V)=\{\begin{array}{lll} c_{t}^{p} & \text{if} & p<p^{*}(t)\\ R_{t}(c,V)-c_{t}^{1}-\ldots -c_{t}^{p^{*}(t)-1} & \text{if} & p=p^{*}(t)\\ 0 & \text{if} & p>p^{*}(t). \end{array}$$

**Figure 3:**
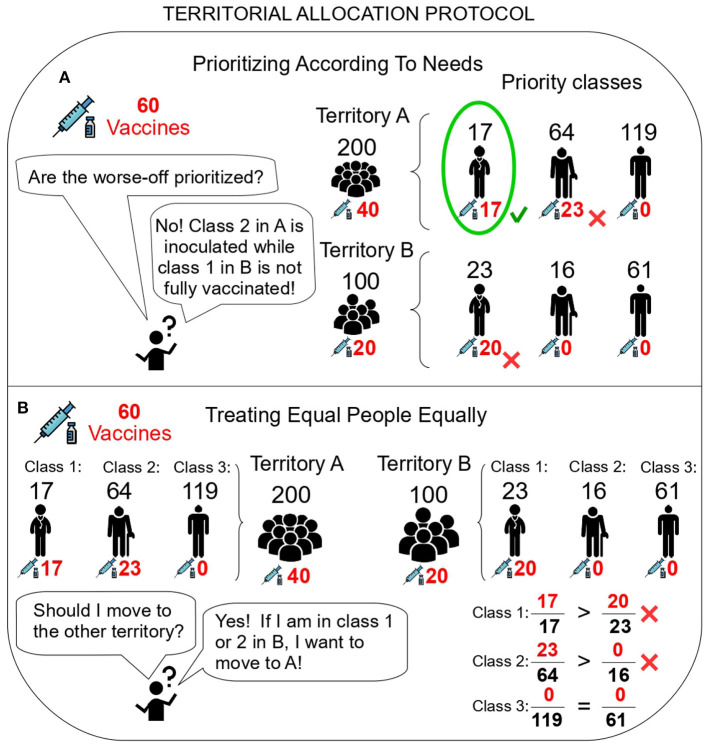
The Territorial Allocation protocol violates ethical principles. **(A)** The Territorial Allocation protocol violates *Prioritizing According To Needs*. Territory A receives two thirds of the vaccinations (40), which suffice to fully immunize the first class and partially inoculate the second ($R_{A}^{1}=17$, $R_{A}^{2}=23>0$). Territory B receives one third of the vaccinations (20), which is insufficient to fully immunize the first class ($R_{B}^{1}=20<23$). **(B)** The Territorial Allocation protocol violates *Treating Equal People Equally*. For the first and second priority classes, vaccination rates are worse in territory *B* than in territory *A*, and hence *B*-residents belonging to those classes would prefer to move to *A*. Icons made by Freepik from Flaticon.

This intuitive protocol violates not one but both of the ethical principles described above. To see this a counterexample suffices. Figure 3 shows an example of Vaccine Allocation Problem where the second priority class in a territory receives vaccinations while the first priority class in another territory is still rationed, hence violating Prioritizing According To Needs (Figure 3A). Further, vaccination rates per class are strictly better in the first territory, violating Treating Equal People Equally (Figure 3B).

### 3.3. Main result

Our main result shows that it is possible to satisfy both ethical desiderata. Strikingly, the axiomatic approach actually pins down the protocol (and the algorithm) which makes this possible. In other words, any other conceivable protocol, currently extant or not, *will* violate one of the principles.

**Theorem 1**. *There exists one and only one Vaccine Rationing Protocol which satisfies Prioritizing According To Needs and Treating Equal People Equally. This is a two-stage procedure as follows:*

• Stage 1: *Allocate vaccinations to priority classes (globally) independently of territories according to priority*.

• Stage 2: *For each priority class, reallocate vaccinations across territories proportionally to the size of the class in them*.

**Figure 4:**
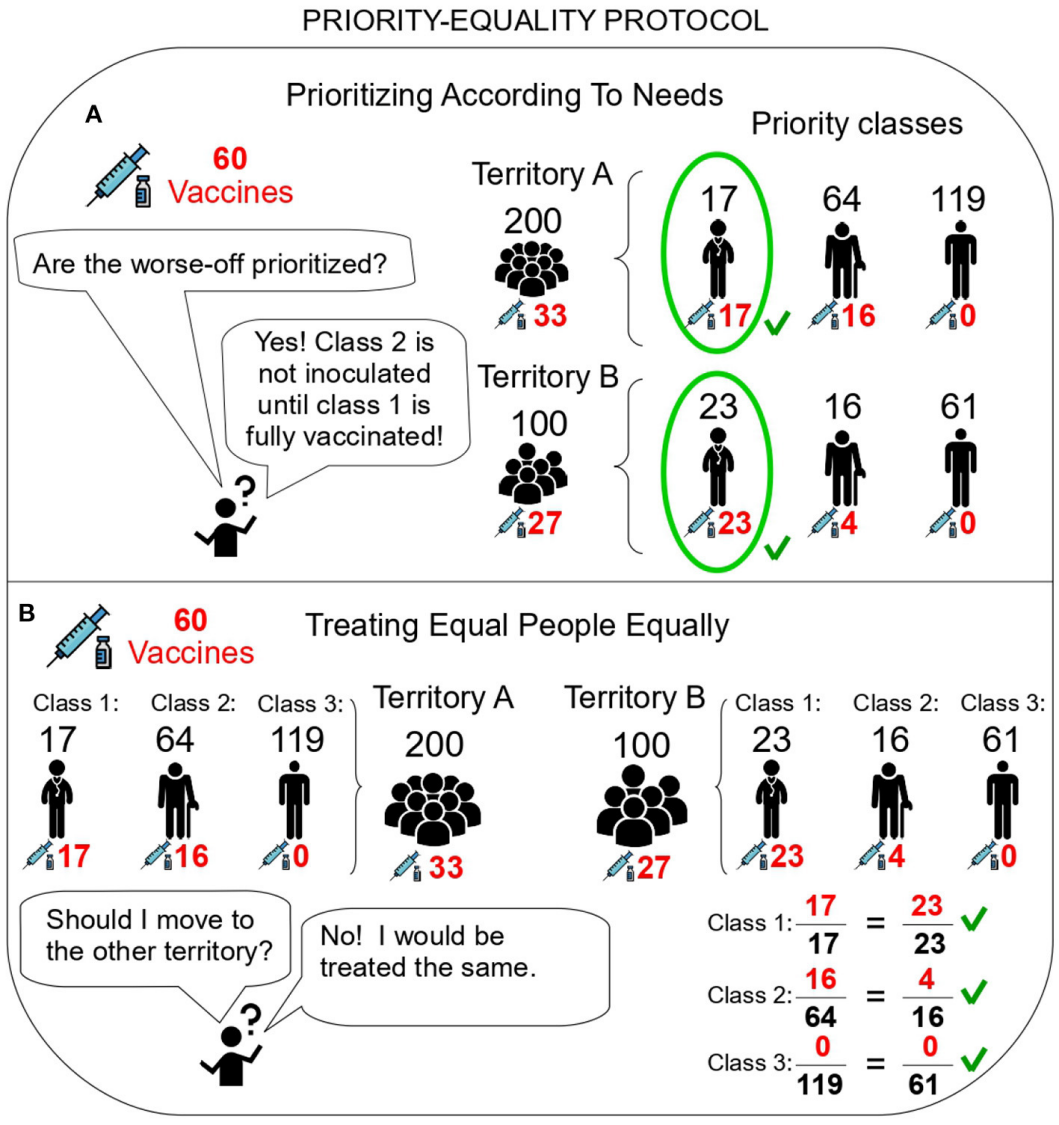
The Priority-Equality protocol satisfies ethical principles. **(A)** *Prioritizing According To Needs*. The highest priority class is fully covered in both territories. The remaining vaccinations (20) do not suffice to cover the second priority class, hence that class is rationed in both territories. Other classes are excluded. **(B)** *Treating Equal People Equally*. For the second class, vaccinations are allocated proportionally to the size of that class across the territories ($R_{A}^{2}=16<64$, $R_{B}^{2}=4<16$). Hence, vaccination rates are identical. Icons made by Freepik from Flaticon.

We refer to this two-stage procedure as the **Priority-Equality protocol** (PE; Figure 2B). Algorithmically, this protocol is as follows (see Figure 4 for an example). In Stage 1, the available vaccinations *V* are distributed following the order of the priority classes (without any distinction across territories). That is, the highest-priority class is vaccinated in all territories, with the remaining vaccinations (if any) going to the second class, and so on until the doses are exhausted. Formally, let p^*^ be the unique class such that *c*^1^ + … + *c*^p^^*−1^ ≤ *V* but *c*^1^ + … + *c*^p^^*^ > *V*, i.e., the first class in order of decreasing priority such that overall available vaccinations do not allow for full coverage. Then, each class *p* is allocated


$$R^{p}(c,V)=\{\begin{array}{lll} c^{p} & \text{if} & p<p^{*}\\ V-c^{1}-\ldots -c^{p^{*}-1} & \text{if} & p=p^{*}\\ 0 & \text{if} & p>p^{*} \end{array}$$

In the second stage, the total allocation to class *p*, *R*^*p*^(*c, V*), is distributed across territories (or, more properly, among their respective *p*-priority classes). This is done proportionally to the territorial claims for each priority class,


$$R_{t}^{p}\left(c,V\right)=\frac{c_{t}^{p}}{c^{p}}R^{p}\left(c,V\right)$$

for the classes with *c*^*p*^ > 0 [if for any class there were no claims, *c*^*p*^ = 0, then $R_{t}^{p}\left(c,V\right)=0$ for all *t*].

Figure 4 illustrates with an example how this protocol fulfills both desiderata. The mathematical proof of our result is provided in Supplementary material A.2. It first shows that the PE protocol fulfills both desiderata for all problems. Crucially, it then shows that any other hypothetical protocol that also fulfills those must be identical to PE.

It is easy to show that the two fundamental ethical desiderata considered here are logically independent (see Supplementary material A.4). Finally, we remark that the PE protocol also fulfills other appealing properties that the TA protocols violate, e.g., robustness to decentralization, equal treatment of territories with identical claims for a given class, or robustness to sequential vaccination campaigns (see Supplementary material A.5).

## 4. Discussion

We offer a possible approach to the problem of allocating scarce vaccines from an ethical perspective. More concretely, we focus on the prominent work “Fair allocation of scarce medical resources in the time of COVID-19” (17). We restrict our attention to this ethical framework for two reasons. First, this is one of the most influential ethical frameworks in the literature. And second, this framework adapts existing, well-known paradigms for the ethical analysis of the allocation of scarce medical resources (16, 18) to the specific crisis of the COVID-19 pandemic. Our contribution consists in converting the ethical desiderata in this framework into formal axioms to analyze their potential compatibility. Our main result is that they are indeed compatible and single out one and only one protocol, which differs from the previously used ones.

The problem we analyze here is of course not limited to the allocation of vaccines during an epidemic. Rather, the protocol is applicable whenever scarce medical resources need to be allocated across different territories or organizations while respecting the ethical principles of prioritization and equal treatment of equals. More generally, the allocation of scarce medical resources is often decided *ad hoc* on the basis of heuristic approaches that often oversimplify the problem (35). The protocol we present here is derived from the formal analysis of the constraints arising from universally-appealing ethical principles, and hence provides a more nuanced approach. Of course, this protocol is not a universal recipe to be blindly applied to every ethical allocation dilemma. Our algorithm is well-suited for the normative allocation of scarce resources whenever three conditions are fulfilled. First, the demand exceeds the resources at hand. Second, administrative records of the patients or resource claimants are available. Third, there is an uncontroversial prioritization of the claimants (as decided by medical experts and the legitimate authority on the basis of scientific evidence). Note that these properties can be fulfilled at multiple levels, from allocations across countries in a supranational alliance or across states in a country to those across healthcare areas in a region or between hospitals in a local network.

The present work is complementary to an important strand of the literature that studies how to allocate priority depending on the societal objective function.^3^ For instance, other studies show that older groups should be vaccinated first if the target is minimizing deaths, but younger groups should be vaccinated earlier if the target is to minimize confirmed cases (37), that the allocation of vaccines among the different sociodemographic groups depends on the stated goal (38), or that infections and economic losses can be minimized combining the optimal vaccine prioritization and the optimal stay-at-home policy (39).

Our approach is, of course, not exempt of limitations. For instance, it has been noted that many rationing methods are too rigid to be applied in very-dynamic crises (40), and this criticism also applies to the protocol we identify. A more important limitation is that, necessarily, our formal approach takes the priority classes *and their priority order* as given. Our paper does not focus on vaccine prioritization, but rather on territorial and class allocation (fulfilling key ethical principles) given the priority classes. Ultimately, the consequences of the protocol might be judged in conjunction with a given definition of priority classes, which is beyond the scope of our contribution. For instance, one might argue that if healthcare workers are prioritized, the protocol implies allocating (relatively) more vaccines to countries that are already in the fortunate situation of having more healthcare workers per capita. This might be problematic from a deontological perspective, since these countries are already better placed to cope with a health crisis. On the contrary, one might counter that this very same fact makes engaging in a global, worldwide supra-national alliance more politically acceptable for the governments of these favored countries, since they are the ones paying the largest share of the bill at the end of the day. Obviously, the protocol we identify will prescribe different allocations depending on the final prioritization decided by the experts in the field. However, our result is orthogonal to these considerations. We only state that, given the priority classes (which are exogenously given and ideally determined by medical experts), there exists one and only one algorithm that allocates vaccines satisfying the ethical desiderata. If a given definition of priority classes is changed on the basis of ethical or political arguments, the ethical principles we discuss still pin down the same algorithm for vaccine allocation across the territories and the new classes.

A further limitation is that our work concentrates on the implications of specific ethical desiderata (17) for allocation procedures. Of course, a myriad of other dimensions are important in practice, ranging from logistic considerations to political constraints. However, it is important to understand that our result is a full characterization. Suppose that one considered some other, further desideratum to be crucial, e.g., arising from political or logistical considerations. Since there is one and only procedure satisfying the two key ethical desiderata, the question of whether there is a procedure satisfying those desiderata and also the additional one is immediately solved: if the PE protocol fulfills that desideratum, the answer is positive. If the PE protocol fails this further desideratum, the answer is negative.

Hence, our result can be seen as either good news or bad news. On the one hand, there exists a procedure fulfilling the fundamental ethical desiderata identified by a wide consensus. On the other hand, there exists only one such procedure, and hence adding any further constraints or wishes is likely to result in an impossibility theorem. We see our result mostly on the positive side. First, it shows that the allocation of scarce medical resources to multi-territorial areas (from medical districts to the whole planet) under priority constraints is feasible while respecting ethical principles. This does not mean that the implementation of such allocations will be logistically simple or politically uncontroversial. The result shows possibility, and hence hope that other difficulties can be overcome. Second, not all possible, additional desiderata will affect the allocation in itself. For example, logistical considerations might impose constraints on which specific vaccine batches can be allocated to which territories, or on the exact timing of delivery. However, if such difficulties can be overcome without affecting the allocation totals derived from the procedure, the properties of the allocation in itself are unaffected. That is, some additional (but of course important) dimensions of the allocation problem might concern implementation and be orthogonal to the properties considered here.

## 5. Conclusion

We show that elementary ethical desiderata can be fulfilled by Vaccine Rationing Protocols. This was by no means a given. The mathematical social sciences are ripe with examples where putting together a number of desiderata results in an impossibility theorem, as (to cite but one example) the celebrated Arrow's impossibility theorem (41) which shows that there exists no democratic voting method fulfilling a number of innocent-looking, ethically-appealing properties.

Our result actually pins down a protocol. If several protocols fulfilled the ethical desiderata, we would have opened Pandora's box and started a discussion about the relative virtues of one or another protocol. On the contrary, there exists only one protocol fulfilling the targeted properties. Using any other protocol, or any variant of PE (no matter how seemingly small the deviation) will result in a procedure which violates either Prioritizing According to Needs or Treating Equal People Equally. Hence, as long as those principles are deemed desirable, the protocol we identify shows the only way to implement them. It is important to note that the protocol is algorithmic and readily implementable. Our analysis provides not a discussion of a framework, but an actual method, down to the specific computations, ready to be implemented out of the box.

Our results lead to immediate recommendations. If decision makers wish to implement the ethical principles reflected by Prioritizing According to Needs and Treating Equal People Equally, currently-used allocation protocols as the Territorial Allocation protocol need to be replaced by the Priority-Equality protocol. This creates the need for territorial authorities to formally agree to the protocol and its implementation. Of course, this will be easier for certain territorial alliances (states within a single country) than others (heavily decentralized multinational entities).

We conclude by remarking that our algorithm is a general tool to allocate any scarce medical resources, including of course vaccines but also newly-developed, expensive treatments, or preventive treatments from a national stockpile (e.g., iodine pills in the face of nuclear threats).

## Data availability statement

The original contributions presented in the study are included in the article/Supplementary material, further inquiries can be directed to the corresponding author.

## Author contributions

CA-F, JG-S, and MG-V conceptualized the project, proposed the formal model, and drafted the paper. CA-F and JG-S revised the manuscript and added examples based on real data. All authors contributed to the article and approved the submitted version.

## Footnotes

^1^Starting in February 2021, Hungary decided to buy Sputnik V and Sinopharm vaccines from Russia and China, respectively (23, 24), considering that the EU scheme was too slow. This helps explain both the high rate of vaccinated healthcare workers on the 12th week of 2021 as well as Hungary's high performance, in Figure 1B, in terms of the classes that each country could have immunized at every point in time along the year 2021. We thank an anonymous referee for this insightful comment.

^2^In the 12th week of 2021, Bulgaria had a rate of only 13.4% fully-vaccinated healthcare workers. However, this rate was still 28.7% in early March 2022, suggesting that Bulgaria's issue is orthogonal to these considerations, and most likely related to the widespread vaccine hesitancy in this country (25–27).

^3^Other works look at more operational aspects of vaccine distribution. For example, the optimal number of doses to hold in an inventory (36).
