# Supplementary material for: Ethical allocation of scarce vaccine doses: The Priority-Equality protocol
Source: Front Public Health. 2022 Dec 13;10:986776. doi: 10.3389/fpubh.2022.986776 (PMC9792380; doi:10.3389/fpubh.2022.986776)
Supplement: Supplementary file 1 [file Data_Sheet_1.PDF]

Supplementary Material: Ethical allocation of scarce vaccine doses: The Priority-Equality protocol

A.1 MATHEMATICAL DEFINITIONS

Fix a population matrix $n = \left[n_t^p\right]_{t=1,\ldots,T}^{p=1,\ldots,P}$, where each n_t^p is a nonnegative integer. A Vaccine Rationing Problem is a pair (c, V) where $c = \left[c_t^p\right]_{t=1,\ldots,T}^{p=1,\ldots,P}$ is a matrix of claims (also nonnegative integers) and $V > 0$ is the number of available vaccinations, such that $0 \leq c_t^p \leq n_t^p$ for all p and t, and $C = \sum_{t=1,\ldots,T}^{p=1,\ldots,P} c_t^p > V$.

Let $\mathcal{V}$ denote the set of all possible Vaccine Rationing Problems. A Vaccine Rationing Protocol is a mapping

$$R : \mathcal{V} \mapsto \mathbb{R}^{P\times T}$$

such that $0 \leq R_t^p(c, V) \leq c_t^p$ for each p and t and

$$\sum_{t=1,\ldots,T}^{p=1,\ldots,P} R_t^p(c, V) = V.$$

The interpretation is that $R_t^p(c, V)$ is the actual number of vaccinations allocated to priority class p in territory t.

A.2 PROOF OF THE MAIN RESULT

We now prove the main result. First, note that PE satisfies Prioritizing According To Needs by construction, because all classes with $p \leq p^*$ receive their full claims, all classes with $p > p^*$ are excluded, and only the class with priority p^* might be rationed. The PE protocol also satisfies Treating Equal People Equally. This is trivial for classes with $p \leq p^*$ (since those are not rationed) and $p > p^*$ (since those are excluded). For class p^*, vaccinations are allocated proportionally, so $R_t^p(c, V)/c_t^p = R^p(c, V)/c^p$ for each territory t with positive claims for p, and the right-hand side of this equation does not depend on t.

Hence, PE fulfills both ethical desiderata. This is already interesting in itself, but the most important part of our result is that *no other protocol* can satisfy those desiderata. To prove this mathematically, suppose that we have some rationing protocol prescribing some allocations $\widetilde{R}_t^p(c, V)$ as a function of the claims c and the total endowment V. We will show that this rationing protocol must actually be PE.

Since $C > V$ and the sum of all allocations $\widetilde{R}_t^p(c, V)$ must equal V, there must exist some rationed classes in some territories. We can distinguish two cases.

Suppose there exists some class p' in some territory t' which is rationed but not excluded, $0 < \widetilde{R}_{t'}^{p'}(c, V) < c_{t'}^{p'}$. Since the protocol fulfills Prioritizing According To Needs, this means that all classes with $p > p'$ must be excluded in all territories, $\widetilde{R}_t^p(c, V) = 0$ for all $p > p'$ and all t. However, by the same reason no class with $p < p'$ can be rationed in any territory, because if it were, we would conclude $\widetilde{R}_{t'}^{p'}(c, V) = 0$, which however is not true.

Recall that the sum of all allocations must be V and $\widetilde{R}_t^p(c,V) \leq c_t^p$. Since classes before p' are not rationed and classes after p' are excluded,

$$c^1 + \ldots + c^{p'-1} + \sum_{t=1,\ldots,T} \widetilde{R}_t^{p'}(c,V) = V$$

(where the initial sum is obviously void if $p' = 1$) and since $\widetilde{R}_t^{p'}(c,V) \leq c_t^{p'}$ for all t and $\widetilde{R}_{t'}^{p'}(c,V) < c_{t'}^{p'}$, we have that the total claims of classes before p' do not exhaust V, but adding the claims of class p' would exceed V. Hence, $p' = p^*$ in the computation of PE, and PE dictates to exclude all classes above p' and not ration classes before p'. That is, the hypothetical protocol $\widetilde{R}$ coincides with PE for all those classes. Further, the total allocation to class $p' = p^*$ is $V - c^1 - \ldots - c^{p'-1}$, as in PE.

By Treating Equal People Equally, $\widetilde{R}_t^{p'}(c,V)/c_t^{p'} = \widetilde{R}_{t'}^{p'}(c,V)/c_{t'}^{p'}$ for any two territories with positive claims for p'. Fix a territory t' with $c_{t'}^{p'} > 0$. We have that

$$\widetilde{R}_t^{p'}(c,V) = \frac{c_t^{p'}}{c_{t'}^{p'}} \widetilde{R}_{t'}^{p'}(c,V)$$

for all t with $c_t^{p'} > 0$. Adding up over all territories, taking into account that $\widetilde{R}_t^{p'}(c,V) = 0$ if $c_t^{p'} = 0$ and substituting this equality we obtain

$$V - c^1 - \ldots - c^{p'} = \sum_{t=1,\ldots,T} \widetilde{R}_t^{p'}(c,V) = \frac{\widetilde{R}_{t'}^{p'}(c,V)}{c_{t'}^{p'}} \sum_{t=1,\ldots,T} c_t^{p'}$$

and hence

$$\widetilde{R}_{t'}^{p'}(c,V) = \frac{c_{t'}^{p'}}{c^{p'}} \left(V - c^1 - \ldots - c^{p'-1} \right)$$

for each territory t' with positive claims for p'. That is, the protocol also prescribes the same as PE for class $p' = p^*$.

The only remaining case is when $\widetilde{R}_t^p(c,V)$ is such that all rationed classes are excluded. Analogously to above, by Prioritizing According To Needs there is a class p'' such that, for all t, $\widetilde{R}_t^p(c,V) = 0$ for all $p > p''$ and $\widetilde{R}_t^p(c,V) = c_t^p$ for all $p \leq p''$. It follows that $p'' = p^*$ in the definition of PE, but in this case the claims of classes $1, \ldots, p''$ exactly exhaust V. Thus the prescriptions of the protocol again coincide exactly with those of PE.

A.3 RELATION TO THE PROBLEM OF ADJUDICATING CONFLICTING CLAIMS

Our formal approach is related to work on the adjudication of conflicting claims (e.g., bankruptcy problems) in the mathematical social sciences. In such problems, an endowment of divisible or indivisible resources has to be distributed among a group of legitimate claimants (1, 2, 3, 4, 5). Vaccine Rationing Problems deal with rationing the available vaccinations at hand among different territories, with individuals belonging to priority classes. Hence, the latter are more complex than the former due to the presence of the priority classes. A series of contributions have considered priority rules in the context of conflicting claims (6, 7, 8), but in these models priority classes emerge endogenously. In contrast, in

Vaccine Rationing Problems priority classes are exogenously decided (on the basis of scientific and ethical considerations).

Applications and expansions of the classical conflicting claims model include normative approaches to the rationing of emissions of greenhouse gases (9, 10), sharing water in transboundary rivers (11), or proportionality rules in financial networks (12). From the formal-analytical perspective, the most-closely related work to Vaccine Rationing Problems as defined here are multi-issue allocation models (13, 14, 15, 16, 17), e.g. when a budget has to be simultaneously allocated to different concepts and different agents. In those models, multi-issue allocations are made in two stages: Stage 1 allocates a resource among the issues and Stage 2 further allocates each issue's resource allotment among the agents. Hence, as in our setting, a matrix of claims results (issues and agents). The main difference is that in multi-issue models no issue needs to be prioritized and there is no relation at all between issues, while in our problems priority classes are related by the priority relation (exogenously dictated by scientific evidence and ethical considerations).

A.4 INDEPENDENCE OF THE PROPERTIES

In this section, we show that Prioritizing According To Needs and Treating Equal People Equally are logically independent, i.e. no property is implied by the other one. To see this, we exhibit two protocols (different from PE and TA), each of which fulfills one of the desiderata but not the other.

Protocol 1 consists on two stages. First, vaccinations are allocated to the priority classes independently of territories according to priority, as in PE. In the second stage, for each priority class, vaccinations are reallocated across territories proportionally to the total claims of each territory. Formally, Stage 1 determines $R^p(c,V)$ as in PE. In Stage 2, for each class p, $R^p(c,V)$ is redistributed across territories (or, more properly, among their p-priority classes) as follows.

$$R^p_t(c,V) = \min\{c^p_t, \lambda \frac{c_t}{C}\}$$

where λ is such that $\sum_{t=1}^{T} \min\{c^p_t, \lambda \frac{c_t}{C}\} = R^p(c,V)$.

It is easy to see that this protocol fulfills Prioritizing According To Needs but violates Treating Equal People Equally.

Protocol 2 also consists on two stages. First, vaccinations are allocated equally (uniformly) among priority classes, unless the claims can be covered. In the second stage, the total allocation to each class p, $R^p(c,V)$, is distributed across territories proportionally to the claims in each priority class, as in Stage 2 of PE. Formally, in Stage 1 one determines

$$R^p(c,V) = \min\{c^p, \lambda\}$$

where λ is such that $\sum_{p=1}^{P} \min\{c^p, \lambda\} = V$.

Then, in Stage 2, each $R^p(c,V)$ is distributed across territories as in PE.

It is easy to see that this protocol fulfills Treating Equal People Equally but violates Prioritizing According To Needs.

A.5 FURTHER PROPERTIES OF THE PRIORITY-EQUALITY PROTOCOL

In addition to the two fundamental ethical desiderata considered in the main text, PE also fulfills a number of other appealing properties which we list here. The first one ensures that equal claims are treated equally across territories.

Equal treatment of equal claims. If several territories make identical claims for a given priority class, they should all receive the same number of vaccinations for that class.

Formally, this desideratum can be expressed as follows. If $c_t^p = c_{t'}^p$ for a given class p and two territories t, t', then $R_t^p(c, V) = R_{t'}^p(c, V)$. This is immediately fulfilled by PE. For all classes that are excluded, the number is zero for all territories. For all classes that are not rationed, every territory receives the claim. For the unique class (if any) which is rationed but not excluded, territories which make the same claim receive the same number of vaccinations since the allocation is proportional to the claims.

Strikingly, this property is violated by protocols as TA, since if one of the territories has a larger population, the first stage of TA will give it more vaccinations independently of the claims. To see a counterexample, suppose territories A and B have populations of 200 and 100, respectively. Both countries are close to full immunization. In each of them, only 80 individuals remain to be vaccinated, all of them in the last priority class. The total number of newly-available vaccinations is $V = 60$. The TA protocol then distributes 40 vaccinations to A and 20 to B ($R_A^3 = 40$, $R_B^3 = 20$). Hence, equal claims would be treated unequally. In contrast, the PE protocol would distribute vaccinations equally ($R_A^3 = 30$, $R_B^3 = 30$).

The second one spells out an immunity to decentralization. For a given vaccination campaign, territories are typically given (nations, states, etc.), However, one could conduct administrative aggregation at a lower level (regions, counties, etc.), or several territories might insist in being treated as a single one. Worse, one nation might insist on one level of aggregation while another uses a different level. The requirement is that the protocol cannot be manipulated in this way, that is, that the result does not differ if territories are split (or merged).

Independence of merging and splitting. If two territories merge, or if one territory splits in two, the protocol keeps the same distribution of doses among priority classes, and other territories are not affected.

Formally, this desideratum can be stated as follows. Let (c, V) and $(\hat{c}, V)$ be two Vaccine Allocation Problems with the same V, the first with T territories and the second with $T + 1$. Suppose that, for all p, $c_t^p = \hat{c}_t^p$ for all $t < T$ and $c_T^p = \hat{c}_T^p + \hat{c}_{T+1}^p$. Then, it must hold that $R_t^p(c, V) = R_t^p(\hat{c}, V)$ for all $t < T$ and $R_T^p(c, V) = R_T^p(\hat{c}, V) + R_{T+1}^p(\hat{c}, V)$.

It is easy to see that PE fulfills this desideratum. Splitting or merging does not affect the total claim per priority class. Thus $R^p(c, V) = R^p(\hat{c}, V)$ for all p. Hence, the classes which can be fully covered, and those that need to be excluded, are not changed at all. For the unique class p^* (if any) that is rationed but not excluded, territories with $t < T$ are unaffected, since their allocation depends only on their claims and the total claim for the class. For the split or merged territories,

$$R_T^p(\hat{c}, V) + R_{T+1}^p(\hat{c}, V) = \left(\frac{\hat{c}_T^p + \hat{c}_{T+1}^p}{c^p} \right) R^p(c, V) = R_T^p(c, V).$$

Again, this is violated by protocols as TA. To see a counterexample, suppose again that territories A and B have populations of 200 and 100, respectively. There are three priority classes and the claims are

$c_A = (15, 45, 140)$ and $c_B = (21, 15, 64)$, but the available vaccinations are $V = 60$. Protocol TA allocates 40 vaccinations to A with a distribution of 15, 25, and 0 for the first, second, and third class, respectively. It also allocates 20 vaccinations to B, all of them to the first class. Suppose that territory A splits into two sub-territories, A_1 and A_2, with claims $c_{A_1} = (11, 35, 74)$ and $c_{A_2} = (4, 10, 66)$, respectively (which add up to the previous claim of A). Protocol TA then recommends 24 vaccinations for A_1 and 16 for A_2, but the distribution in $A1$ is 11, 13, and 0 for the first, second, and third priority class, respectively, and in A_2 it is 4, 10, and 2. Thus A_1 and A_2 together receive 23 vaccinations for the second class and 2 for the third, while before the split they received 25 for the second and none for the third. Hence the protocol's results depend on the administrative divisions.

The last property we consider refers to dynamic allocations. Suppose we consider the problem of vaccine allocation as a dynamic one where different batches arrive in time, i.e. V' vaccinations are available after an initial V have already been used. The requirement is that applying the protocol to each batch separately produces the exact same result as considering the whole problem anticipating that $V + V'$ vaccinations will be eventually available.

Invariance Under Sequential Vaccination. If V vaccinations are available earlier and V' are available later, applying the protocol dynamically (i.e. separately to each batch) yields the same results as applying it statically to the total batch $V + V'$.

Formally, this requirement means that considering Vaccine Allocation Problems (c, V) and $(c, V + V')$ with $V, V' > 0$, it must hold that $R_t^p(c, V + V') = R_t^p(c, V) + R_t^p(c - R(c, V), V')$ for each p and t, where $c - R(c, V)$ are the remaining claims after $R(c, V)$ has been applied, i.e. class p in territory t has the remaining claim $c_t^p - R_t^p(c, V)$.

It is easy to see that this is fulfilled by PE, because the unique class (if any) which is rationed but not excluded given $V + V'$ is the same as when $R(c, V)$ has already been applied and then V' vaccinations become available.

REFERENCES

[1]O'Neill B. A problem of rights arbitration from the Talmud. *Mathematical Social Sciences* **2** (1982) 345 – 371.

[2]Aumann R, Maschler M. Game theoretic analysis of a bankruptcy problem from the Talmud. *Journal of Economic Theory* **36** (1985) 195–213.

[3]Moulin H. Axiomatic cost and surplus-sharing. Arrow K, Sen A, Suzumura K, editors, *Handbook of Social Choice and Welfare* (Elsevier) (2002), vol. 1, 289–357.

[4]Thomson W. *How to divide when there is not enough: From Aristotle, the Talmud, and Maimonides to the Axiomatics of Resource Allocation*, vol. 62 (Cambridge University Press) (2019).

[5]Estañ T, Llorca N, Martínez R, Sánchez-Soriano J. On the Difficulty of Budget Allocation in Claims Problems with Indivisible Items and Prices. *Group Decision and Negotiation* **30** (2021) 1133–1159.

[6]Moulin H. Priority rules and other asymmetric rationing methods. *Econometrica* **68** (2000) 643–684.

[7]Flores-Szwagrzak K, García-Segarra J, Ginés-Vilar M. Priority and proportionality in bankruptcy. *Social Choice and Welfare* **54** (2020) 559–579.

[8]García-Segarra J, Ginés-Vilar M. Additive adjudication of conflicting claims. *International Journal of Game Theory* (2022).

[9]Ju BG, Kim M, Kim S, Moreno-Ternero JD. Fair international protocols for the abatement of GHG emissions. *Energy Economics* **94** (2021) 105091.

[10]Duro JA, Giménez-Gómez JM, Vilella C. The allocation of CO2 emissions as a claims problem. *Energy Economics* **86** (2020) 104652.

[11]Estévez-Fernández A, Giménez-Gómez JM, Solís-Baltodano MJ. Sequential bankruptcy problems. *European Journal of Operational Research* **292** (2021) 388–395.

[12]Csóka P, Herings PJJ. An axiomatization of the proportional rule in financial networks. *Management Science* **67** (2021) 2799–2812.

[13]Calleja P, Borm P, Hendrickx R. Multi-issue allocation situations. *European Journal of Operational Research* **164** (2005) 730–747.

[14]Lorenzo-Freire S, Casas-Méndez B, Hendrickx R. The two-stage constrained equal awards and losses rules for multi-issue allocation situations. *Top* **18** (2010) 465–480.

[15]Bergantiños G, Lorenzo L, Lorenzo-Freire S. A characterization of the proportional rule in multi-issue allocation situations. *Operations Research Letters* **38** (2010) 17–19.

[16]Bergantiños G, Lorenzo L, Lorenzo-Freire S. New characterizations of the constrained equal awards rule in multi-issue allocation situations. *Mathematical Methods of Operations Research* **74** (2011) 311–325.

[17]Bergantiños G, Chamorro JM, Lorenzo L, Lorenzo-Freire S. Mixed rules in multi-issue allocation situations. *Naval Research Logistics (NRL)* **65** (2018) 66–77.
